# Atmospheric Biodetection Part I: Study of Airborne Bacterial Concentrations from January 2018 to May 2020 at Saclay, France

**DOI:** 10.3390/ijerph17176292

**Published:** 2020-08-28

**Authors:** Roland Sarda-Estève, Dominique Baisnée, Benjamin Guinot, Gediminas Mainelis, John Sodeau, David O’Connor, Jean Pierre Besancenot, Michel Thibaudon, Sara Monteiro, Jean-Eudes Petit, Valérie Gros

**Affiliations:** 1 Laboratoire des Sciences du Climat et de l’Environnement, LSCE/IPSL, Unité mixte de recherche CEA-CNRS-UVSQ, 91190 Saint-Aubin, France; dominique.baisnee@lsce.ipsl.fr (D.B.); jean-eudes.petit@lsce.ipsl.fr (J.-E.P.); valerie.gros@lsce.ipsl.fr (V.G.); 2 Laboratoire d’Aérologie, Université Toulouse III, CNRS, UPS, 31400 Toulouse, France; benjamin.guinot@aero.obs-mip.fr; 3 Réseau National de Surveillance Aérobiologique, 69690 Brussieu, France; jeanpierre.besancenot@orange.fr (J.P.B.); michel.thibaudon@wanadoo.fr (M.T.); 4 Department of Environmental Sciences, School of Environmental and Biological Sciences, Rutgers, The State University of New Jersey, New Brunswick, NJ 08901-8525, USA; mainelis@envsci.rutgers.edu; 5 Department of Chemistry and Environmental Research Institute, University College Cork, T12 YN60 Cork, Ireland; j.sodeau@ucc.ie; 6 School of Chemical and Pharmaceutical Sciences, Technological University of Dublin, D06F793 Dublin 6, Ireland; 453623@dit.ie; 7 Themo Fisher Scientific, 18 avenue de Quebec, 91941 Villebon Courtaboeuf, France; Sara.Monteiro@thermofisher.com

**Keywords:** bacteria, cultivable method, flow cytometry, source-receptor model, geographical origin

## Abstract

*Background:* The monitoring of bioaerosol concentrations in the air is a relevant endeavor due to potential health risks associated with exposure to such particles and in the understanding of their role in climate. In this context, the atmospheric concentrations of bacteria were measured from January 2018 to May 2020 at Saclay, France. The aim of the study was to understand the seasonality, the daily variability, and to identify the geographical origin of airborne bacteria. *Methods:* 880 samples were collected daily on polycarbonate filters, extracted with purified water, and analyzed using the cultivable method and flow cytometry. A source receptor model was used to identify the origin of bacteria. *Results:* A tri-modal seasonality was identified with the highest concentrations early in spring and over the summer season with the lowest during the winter season. Extreme changes occurred daily due to rapid changes in meteorological conditions and shifts from clean air masses to polluted ones. *Conclusion*: Our work points toward bacterial concentrations originating from specific seasonal-geographical ecosystems. During pollution events, bacteria appear to rise from dense urban areas or are transported long distances from their sources. This key finding should drive future actions to better control the dispersion of potential pathogens in the air, like persistent microorganisms originating from contaminated areas.

## 1. Introduction

Atmospheric Primary Biological Aerosol Particles (PBAPs) like pollen, fungal spores, bacteria and viruses affect climate and have an impact on human health. For example, they can modify the hydrological cycle due to their microphysical and chemical properties. They are also involved in causing human and animal diseases due to their pathogenicity [1,2,3,4,5]. This is particularly true for airborne bacteria which can act as Ice Nuclei (IN) [6,7,8,9], and induce severe diseases like pneumonia or tuberculosis, when deposited in the upper respiratory tract [10,11,12]. One of the best-known airborne disease agents is bacterium *Bacillus anthracis*. Despite its low occurrence (less than 5% of bacteria found in the air), its mortality rate in the absence of treatment is up to 90 and even 100% [13]. Due to their pathogenicity, bacteria are often monitored in various indoor environments like farms, workplaces, houses, classrooms, public hospitals, and space habitats [14,15,16,17]. Long-term studies reporting outdoor observations of bacterial concentrations are much scarcer in number, as they require a lot of logistics and consumables, and are labor intensive. Data and knowledge are, therefore, insufficient in this domain. Several climate-related studies have been performed over several months to study the PBAPs seasonality, to understand the Cloud Condensation Nuclei (CCN) and Ice Nuclei (IN) properties or biodiversity, or to investigate the impact of agricultural activities and waste industry on the environment. Therefore, due to the multi-modal relevance of this multidisciplinary science, ranging from observations to modeling, the investigation of long-term atmospheric bacterial concentrations is needed [18,19,20,21,22,23,24]. Their seasonal biodiversity is also important to understand as it allows us to determine their geographical origin and linkage to a specific source, and also allows us to evaluate the dispersion processes involved [25,26,27]. This last point could explain the occurrence of particular diseases within a specific ecosystem, in particular for bacteria and virus due to their long redsidence time in the atmosphere [28].

In this context, different approaches have been used in the literature to collect and analyze the bacterial concentrations in the air based on filtration, wet collectors, or electrostatic precipitation [29,30,31,32,33,34]. Recently, in order to investigate the hourly variability of PBAPs, online measurements by fluorescence methods have been developed and used in different environments [35,36]. Additionally, online detection and collection with automated wet cyclones were used to avoid culture and laboratory work for species identification [37]. To date, these fluorescent methods have limitations and suffer from artifacts without providing a clear characterization of the bioaerosol. At the same time, the literature points out to a clear need to study the daily variability of PBAPs, and in particular bacteria, to (1) understand what the key parameters driving their concentration in the air are; (2) localize their geographical origins and characterize their transport pattern during pollution events; and (3) assess their impact on the environment using different modeling approaches [38,39]. This article presents long term measurements of cultivable bacteria, the meteorological factors controlling their variability in relation with pollen and fungal spores and their geographical origins in Saclay, a suburban area of Paris. The results previously obtained from this site for pollen and fungal spores demonstrated the relevance of the use of an onsite source model receptor, using local meteorological data [40,41,42].

## 2. Materials and Methods 

In this study, we have developed a methodology to collect airborne bacteria by filtration, in order to determine their atmospheric concentration in the ecosystem of Saclay. We used the cultivable technique as a first approach to characterize their seasonality. We also started to develop a method by flow cytometry to have ancillary measurements to aid in the estimation of total atmospheric bacteria concentration, which includes culturable, nonculturable, dead, or live bacteria. Finally, we used a source model receptor to identify the geographical origin of airborne bacteria impacting the observation site and validate our hypothesis. 

### 2.1. Description of the Observation Site

**Figure 1:**
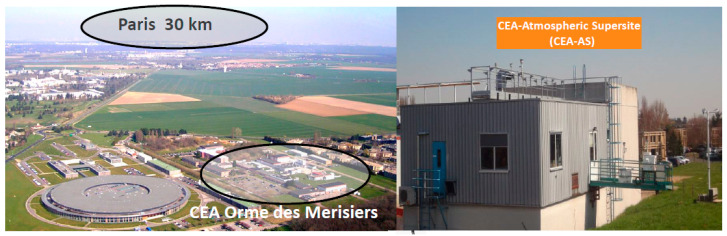
The geographic location of the Saclay EU-ACTRIS Observatory.

The Saclay SIRTA observatory is part of the European Research Infrastructure for the observation of Aerosol, Clouds, and Trace Gases (EU-ACTRIS) made of observing stations and exploratory platforms. The monitoring site is located at Saclay, France (48.7247° N, 2.1488° E), an oceanic degraded environment impacted by marine and continental air masses, approximately 30 km in the southwest of Paris Megacity. It offers different atmospheric regims like background levels with oceanique air masses and continental processed air masses. Moreover, the observatory is regularly impacted by the polluted plume of Paris. The station stands on the top of a building 15 m above the ground. Since 2014, it has been included in the French Network of Aerobiology Monitoring (Réseau National de Surveillance Aérobiologique, RNSA). In 2014, it hosted the first international intercomparison on bioaerosol detection methods intercomparison described in [41,42]. As illustrated by Figure 1, the sampling site stands in a flat semi-rural area surrounded by crops, forest, and small residential villages. All the standardized sampling inlets and instruments are installed on the roof. The observatory provides full data sets of aerosols, bioaerosols, gases, and meteorological parameters. The site is referenced as an ACTRIS topics center for the Aerosol Chemical Monitoring Calibration Center (ACMCC) and all the details can be found in [43] and the references therein. 

### 2.2. Collection and Sample Analysis

This section presents the protocol used to study airborne bacterial concentrations. Daily samples were collected from the 10th of January 2018 to 31th of May 2020 which represents 880 samples. It is well known that most bacteria in natural environments exist in a viable, although non-cultivable state; 1% to 1.5% of the bacteria are thought to be cultivable. Therefore, it is difficult to estimate bacterial concentrations in the air with culture methods alone. Nevertheless, such methods do not affect the variability or biodiversity and provide robust data sets when it is associated with other analytical techniques such as flow cytometry and DNA analysis [44,45,46,47,48,49,50]. 

#### 2.2.1. Collection, Extraction and Culture Procedure

During the different BIOaerosol DETECTion (BIODETECT) campaigns, from 2014 to 2018, several methods and types of filters were tested to collect airborne bacteria by filtration. The objectives of these campaign in collaboration with the French Network of Aerobiology Monitoring was to evaluate all the techniques which provide online detection, discrimination of bioaerosols present in the air and their IN properties describe in [41,42] and the reference therein. Assuming bacteria are attached to atmospheric Particulate Matter (PM) [51], the results pointed out that the best compromise between collection duration and extraction efficiency adapted to the culture method was to use polycarbonate filters instead of gelatin filters, to avoid desiccation during the 24-h sampling [52]. 

**Figure 2:**
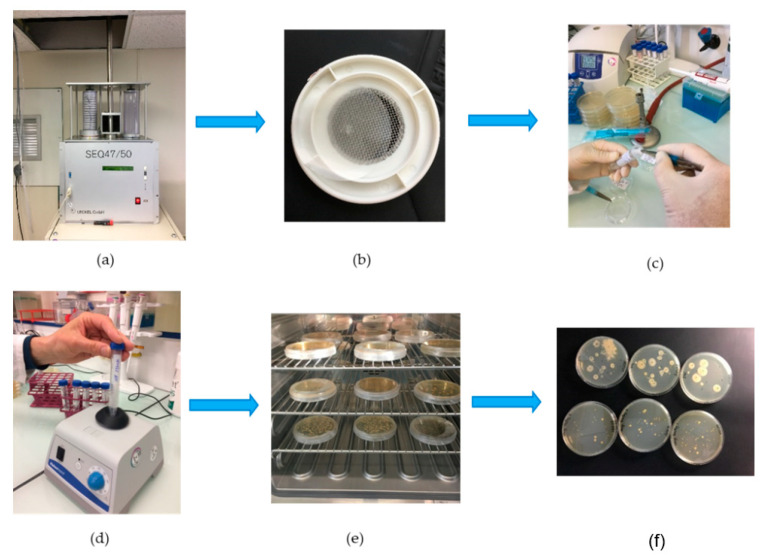
From atmospheric bacteria collection to cultivable counting: (**a**,**b**) sampling, (**c**) recovery of the filtrates in 3 mL of ultra-pure water, (**d**) extraction by stirring, (**e**) seeding on selective nutrient medium and incubation, (**f**) counting of colonies.

Bacteria were captured using an automatic sequential sampler SEQ47/50 from LECKEL (Berlin, Germany), equipped with magazines loading up to 17 filter holders. This sampler is designed for outdoor and indoor uses at all temperatures. This instrument complies with the PM10 and PM2.5 sampling standard CEN EN 12341. It was installed indoors just beneath the roof. The inlet line was a 3-m long stainless-steel tube for the collection of airborne particulate matter following the standard CEN EN 14907. The head was capped with a standardized stainless type protection inlet, to prevent rainwater from entering, and ran without a pre-selector in order to sample the Total Suspension Particulate Matter (TSPM). The outdoor air was sampled at a flow rate of 2.3 cubic meters per hour (38 L/min). The atmospheric particles were filtered every 24 h, from 00:00 to 23:59, on a 47-mm polycarbonate membrane with a porosity of 8 microns (Nuclepore™ Whatman, France). The filtration surface on a 47-mm diameter filter placed in this type of filter holder is 1134 mm^2^, and the calculated Face Velocity (FV) is 56.3 cm/s. The cut-off diameter has been estimated to be 0.8 +/− 0.2 µm following earlier studies of [53,54]. Before refilling with new filters, filter holders and magazines were cleaned with ethanol and dried at room temperature. To control possible contaminations in the magazine, a field blank was made every 15 days in and out from the filter holders. Samples were removed once a week, and the membranes were removed from the filter holders and placed in sterile 10 mL Falcon™ centrifuge tubes (Corning Life Science, Villebon Courtaboeuf, France). The dissolution of the particulate matter was done by adding 3 mL of ultrapure water treated with UV (Thermo Scientific, Smart2 Pure 6 UV/UF, France), while the extraction was performed by vortexing for 1 min at 3000 Rotations Per Minute (RPM), using a Vortex stirrer (Fisherbrand™, ZX3, Villebon Courtaboeuf, France). An aliquot of 100 µL was spread through a disposable sterile inoculation loop (Thermo scientific, Steriline™, Villebon Courtaboeuf, France) on a 90-mm-diameter Petri dish, filled with 30 mL of a nutritive culture medium made of Tryptone Soy Agar (TSA) (Bacterial Flora, FLB, S.A.S Axbiotec, Saint Clément les places, France) and an anti-fungal agent at a concentration of 1% per volume (Amphotericin B, Sigma-Aldrich Chimie Sarl, St. Quentin Fallavier, France) in order to inhibit yeast and mold growth. All the inoculation operations were done in positively pressurized laboratory space and close to the sterile zone provided by a Bunsen burner. The inoculated Petri dishes were placed in a bacteriological incubator (Memmert IF55+, Villebon Courtaboeuf, France) set at 32 °C (+/− 1 °C) to provide the optimal growth conditions for atmospheric bacteria reported from environmental observations [55,56,57]. Colony Forming Units (CFU) counts were performed at 24 and 48 h using a colony counter pen (VWR, Counter Pen™, Fontenay sous bois, France). The complete procedure is illustrated in Figure 2.

Atmospheric concentrations and standard deviation of airborne cultivable bacteria were calculated after field blanks subtraction and sample reproducibility studies using the same culture media. During the period of observation (from 10 January 2018 to 31 May 2020), 79 field blanks have been analyzed: 73% of the blanks presented no contamination and 27% were positive where 17% with one colony, 5% with two colonies and 5% with three colonies. Reproducibility was examined on 28 inoculations from the same sample, at the same time, and led to a mean concentration of 10 colonies ± 2.6 colonies, following the Pearson Standard Deviation Calculation. Overall, it represented a bias of 26% in the growing process. Thus, the results obtained for the blanks indicate that the blanks were less of an influence on the final results compared to the bias (e.g., random variability) due to plate spread and growing processes. Those results give confidence in the sampling procedure established, and support that no external contamination from the filters holders and magazines occurred. The overall uncertainty on the concentrations for our observations was admitted to be ±26% colonies on counting.

#### 2.2.2. Flow Cytometry Analysis of Atmospheric Samples

Flow cytometry is an instrumental technique which measures several optical and physical parameters of particulate matter present in liquid suspension and excited by a laser beam. The technique is widely used in medical and bacteriological laboratories to simultaneously analyze the size and granularity of biological cells like bacteria in water or more complicated mixtures (soils, lakes, seawater). The methodology is well described in the literature [58,59]. Briefly, this instrument is composed of a microfluidic part to separate the particles present in a liquid flow, then, to place them in a small detection chamber where they are excited by a laser beam. Light scattering and fluorescence from the biological material are eventually measured by an optical system composed of photomultipliers. The use of different types of dyes allows differentiating the population present in the sample according to different particle types, hence refining the results. The different physical parameters allow the flow cytometry technique to be able to precisely segregate a complex mixture of bioparticles by size and by type, in a short time. The use of cell-permeating dye is generally used to label bioparticles and bacteria [60,61,62]. In our study, we have applied this technique to calculate total bacterial concentrations in atmospheric samples (Attune NxT, Thermofisher Scientific, Villebon Courtaboeuf, France). After the extraction of our samples, described in Section 2.2.1, an aliquot of 500 µL was taken and filtered through a 5 µm filter to remove big particles of non-interest and preserve the bacterial community which is typically in the range of 0.5 and 2 microns, sometimes up to 5 µm when aggregated in the air [63]. Each sample was stained for 15 min with the nucleic-acid-binding dye SYTO 9 (SY9) Live/Dead BacLight Bacterial Gram Assay 0.1% of SY9 (S34854, ThermoFisher Scientific, Villebon Courtaboeuf, France) and stirred before injection. To get a better particle separation and the best possible sensitivity, a fraction of 100 µL out of the 500 µL was analyzed three times at 25 µL/min.

To accurately quantify the concentration of total bacteria in our samples, we developed a methodology of eight steps that deals well with the major inconvenience of this technique: the need to isolate the background from the sample signal, particularly at low concentrations of bacteria. The complete procedure is summarized in Table 1.

**Figure 3:**
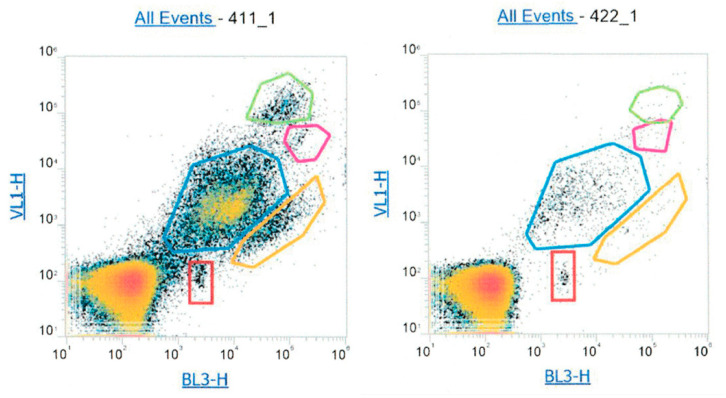
Microbial population distribution across unfiltered sample N411 (24 February 2019) and N422 (7 March 2019) were where VL1-H (440/50) is the fluorescence emission from violet (405 nm) laser, and BL3-H (695/40) is the fluorescence emission from the blue laser (488 nm). The combination of these 2 fluorescence emission detectors gave the broadest population distribution.

The flow cytometry technique is widely used to detect viable microorganisms in different matrices, and particularly in drinking water. Our atmospheric samples were extracted in pure water and led to 3 mL of the liquid sample containing organic, inorganic, soluble, and insoluble particulate matter [54]. We assumed that the sample can be considered similar to rain in terms of osmotic pressure; thus, the potential for osmotic shock is considerably reduced, and (1) avoids breaking of the microorganism cells, while (2) preserves a maximum of viability and particularly cultivable bacteria. The use of this technique in our sample allowed us to estimate the total atmospheric bacterial concentrations without differentiating the metabolic conditions of the microorganisms. The results of the first experiments showed that the unfiltered atmospheric samples were extremely complex in terms of size, fluorescence, and microbial populations. The geometrical gates which describe the populations found in the sample are often named by the letter R and associated with a number and a color; here, five gates were identified as illustrated by Figure 3 and reported by the authors of [64].

**Figure 4:**
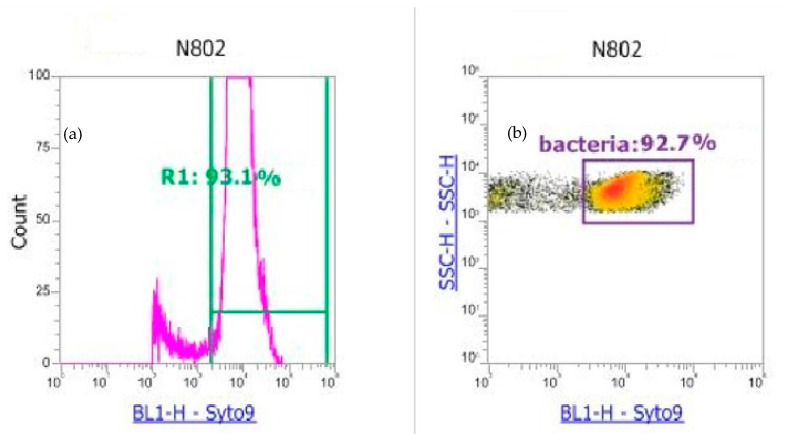
Determination of the bacteria detection zone R1 (**a**) and (**b**) the associated population counting isolated by the R1 gate, were SSC-H—SSC-H is the size scatter and BL1-H the fluorescence induced by the blue laser.

**Figure A1:**
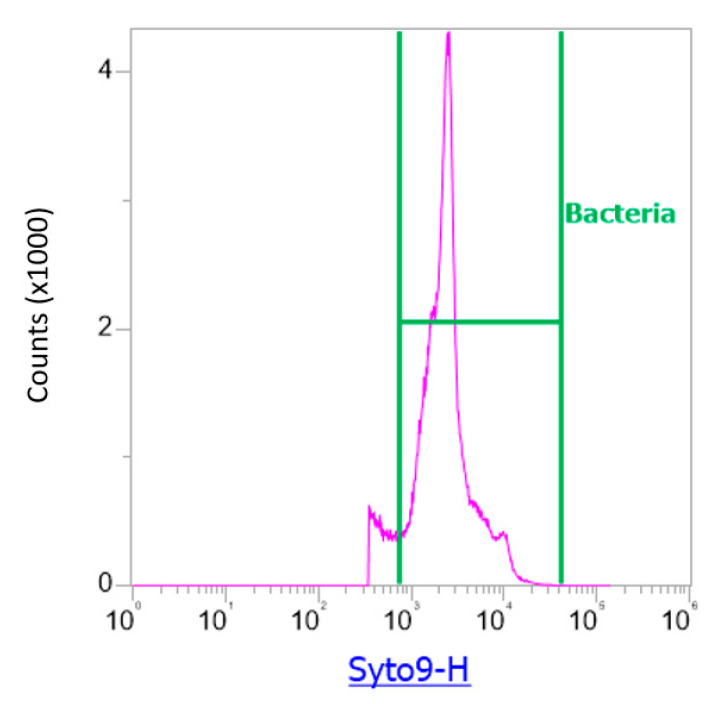
Determination of the bacteria detection zone R1 (green frame) in full size where Syto9-H is the range in the fluorescence signal.

During the optimization procedure, unfiltered and filtered samples were compared based on different syringe pore sizes (from 0.1 to 5 µm) and validated with an injection of a mixture of atmospheric bacteria obtained though the culture technique. Different single bacteria were injected to validate the detection and syringe filtration procedure for the definitions of the gates, as illustrated by Figure 4 and Figure A1.

To reduce the uncertainty on the identification and focus on the total bacterial concentrations, all the samples were filtered with 5-µm syringe filters to preserve and highlight the bacteria signal. The SY9 marker was added to aid in the separation of viable and non-viable bacteria counts [61]. This approach has been used by the authors of [48] to reduce the potential interferences to quantify bacteria in atmospheric samples. To cover a large range of concentration and matrix complexity, the optimization process was achieved on the filters collected during a winter pollution episode.

**Figure 5:**
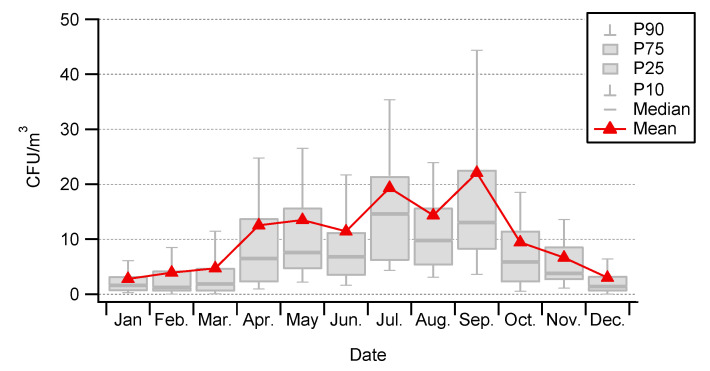
Seasonality of airborne cultivable bacteria at Saclay from 10 January 2018 to 31 May 2020.

When comparing the cultivable method to the counting one, the best results for the estimation of total bacterial concentration arose for the R3 yellow gate (Figure 5), and the population identified in our samples is limited to this area. Therefore, the flow cytometry technique can be used to estimate total bacteria in atmospheric samples after a complete optimization, from the injection procedure to data treatment. The result obtained showed that, in our case, the cultivable technique does correlate with the total bacterial concentrations present in the air, depending on viability and the diversity present in the sample. The losses have been estimated to be in the range of 76.8% to 99.2% (Table A1) by comparing the counts obtained with the cultivable method and the counts obtained with the flow cytometry method with R3 gate. Our results are in accordance with previous reports made for different matrices [45]. The atmospheric bacterial concentrations obtained in our case study, using the flow cytometry, are in accordance with observations performed in other locations [65,66]. However, to date, observations mainly reflect large bacteria since some bacteria counts are still missing due to the sample complexity and the presence of very small bacteria or spores present in the background noise of flow cytometry. Therefore, refinement in the method has to be considered in future works.

At this stage of the study, we know that the bacterial atmospheric concentrations are underestimated due to the method: stress during filtration, resulting in a loss of cultivability. We start to analyze the samples with the flow cytometry technique to have a first comparison point before applying the 16sRNA method. The latter will be presented in a separate paper.

#### 2.2.3. Geographical Origins of Total Airborne Bacteria Affecting Saclay

By coupling the ambient concentrations of bioaerosols with onsite measured wind data, it is possible to retrieve the geographical origins of bacteria reaching the Saclay observatory. Meteorological parameters were provided by the weather station WXT520 (Vaisala, Vantaa, Finland). The measurements of Wind Speed (WS, km/h), Wind Direction (WD, Degrees), Temperature (T, °C), Relative Humidity (RH, %), cumulative rain (R, mm) were acquired every minute. We considered a variant of the two-dimension Non-parametric Wind Regression (NWR), originally developed by the authors of [67], then later adapted by the authors of [68] and designated as the Sustained Wind Incidence Method (SWIM). Previous works have successfully applied this method to determine the geographical origins of pollen and fungal spores [41,42]. Briefly, this variant of NWR takes into account, on a daily basis, the standard deviation of the wind speed and the wind direction.

Equation (1) describes the calculation of SWIM ($S_{i}$):

(1) $$S_{i}=\frac{C_{i}\cdot \Upsilon_{i}}{\max\left(C_{i}\cdot \Upsilon_{i}\right)}\cdot \frac{\delta }{\delta_{i}}$$

where C_i_, Υ_i_, and δ_i_, respectively, represent wind speed, wind direction, and wind direction standard deviation. This actually allows downwind daily concentration values associated with high atmospheric variability to be obtained during that day. Wind direction standard deviation was estimated by the 1-pass Yamartino equations [69]. This entire study was performed with ZeFir-v3.7, a user-friendly tool for wind analysis available online: https://sites.google.com/site/ZeFirproject.

## 3. Results

### 3.1. Seasonality of Cultivable Bacterial Concentrations at Saclay

Interestingly, three modes in the seasonal cycle were evident in our results. The minimum concentrations of cultivable bacteria were found in winter conditions, from December to the end of March (5 ± 1 CFU/m^3^, on average). The first peak appears in April–May (14 ± 3 CFU/m^3^, on average) and a second peak in July (19 ± 4 CFU/m^3^, on average) and a third one in September (22 ± 5 CFU/m^3^, on average). Noticeably, concentrations suddenly dropped in October–November (5 ± 1 CFU/m^3^, on average), as shown in Figure 5.

### 3.2. Daily Variability of Bacterial Concentrations at Saclay

**Figure 6:**
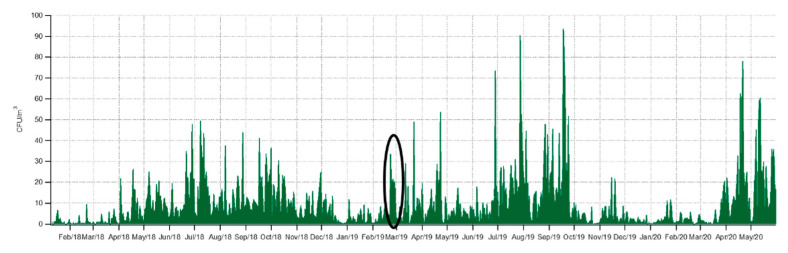
Daily variability of airborne cultivable bacteria at Saclay. The dark circle highlights the pollution episode from 21 February 2019 to 24 February 2019.

**Figure A2:**
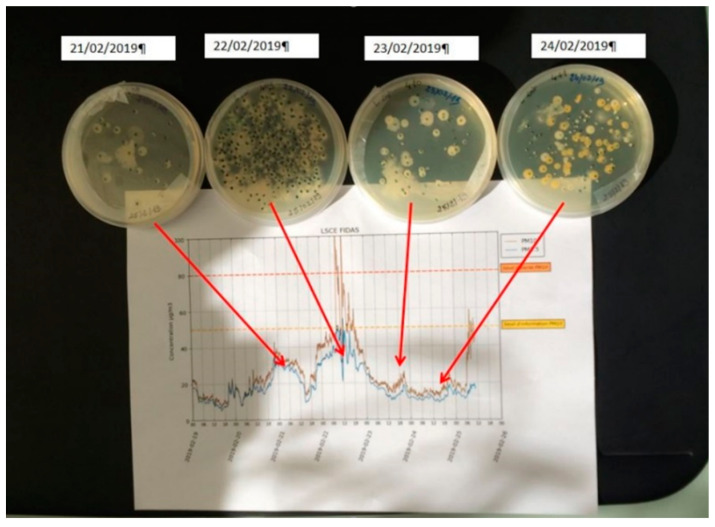
Colors and Shape of different culturable bacteria obtained at Saclay from different air masses.

Over the study period, we observed that the cultivable bacterial concentrations at Saclay could be extremely variable from day to day, as presented in Figure 6. Strong daily variations were particularly observed during the spring and summer seasons. For example, in 2018, concentrations varied from 2 ± 1 to 22 ± 6 CFU/m^3^ between 2nd and 3rd April, respectively, and from 5 ± 1 to 50 ± 13 CFU/m^3^ between 7th July and the day after; that is a 10-fold increase in both cases. Similar daily variability was also observed in 2019 (Figure 5). Surprisingly, a 7-day period revealed an interesting pattern during winter 2019. A jump in daily bacterial concentrations was observed from 21th to 28th February: from 4 ± 1 CFU/m^3^ the first day, to 34 ± 10, 19 ± 5, 21 ± 5, 22 ± 5, 19 ± 5, 21 ± 4, 7 ± 2 CFU/m^3^ the following days, respectively. The concentrations were indeed significantly above the mean value for a winter period of 5 ± 1 CFU/m^3^. Moreover, a change in the shape and color was also observed and correlated with a continental pollution event impacting the observatory. This pollution event displayed PM10 and PM2.5 concentrations above the standard European daily limits as illustrated by Figure A2. The chemical composition of PM1 concentrations was characterized by an Aerosol Chemical Speciation Monitor (ACSM), technical details in [43] and the references therein. The instrument which was located at the observatory was indicating that a plume of pollution from Paris was just followed by continental airmasses affecting the observation site.

Those observations made at Saclay are in accordance with those previously reported elsewhere, performed for short periods of time, and with a daily resolution only [70]. Therefore, further investigations have been undertaken as to the best of our knowledge, the work here represents the first data set with a continuous and daily resolution of bacterial concentrations over a multi-year period. Moreover, during spring 2020 the concentrations increased by a factor 2.5 in average compared to 2018 and 2019 due to exceptional hot weather conditions in France.

### 3.3. Geographical Origin of Bacterial Concentrations

**Figure 7:**
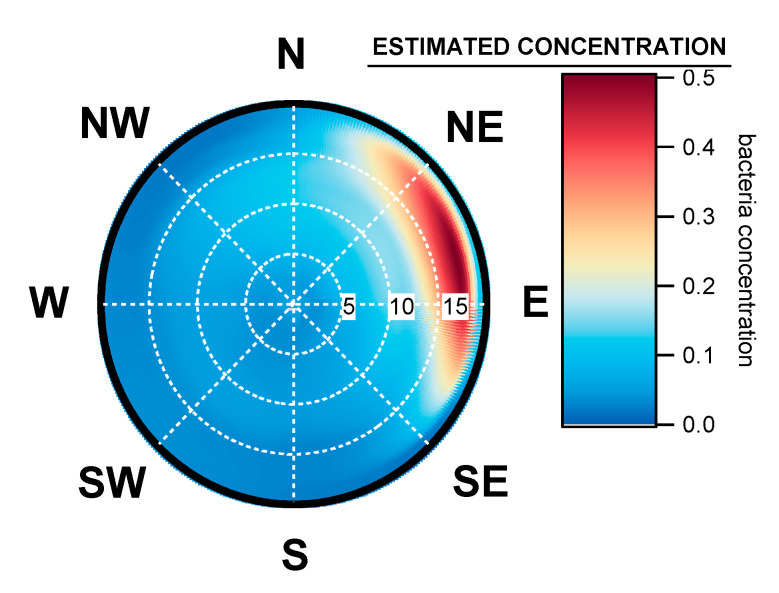
Main geographical origin of bacterial concentration impacting Saclay observatory. The dotted white circles represent the wind speed scale in kilometer per hour (km/h), and the color grid represents the origin of the highest estimated concentration for any wind speed and wind direction.

**Figure A3:**
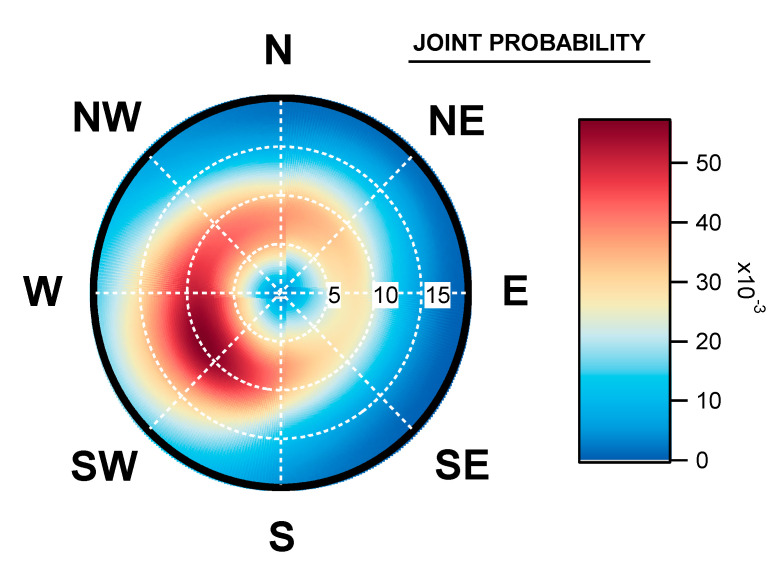
Prevailing wind directions affecting the Saclay observatory during the observation period from 10 January 2018 to 31 May 2020.

The 2-year dataset computed using ZeFir showed that the bacterial concentrations measured at Saclay mainly originated from a large wind sector ranging from North-North East (NNE) to East-South East (ESE). A large point source was identified within the ENE sector, associated with moderate wind speeds between 12 km/h and 17 km/h, as shown by Figure 7. Neither the prevailing South-West (SW) winds, nor the “wet” West (W) sector, which mostly carries maritime air masses, appeared to not influence the atmospheric bacteria loads (Figure A3).

## 4. Discussion

The simplicity of the used experimental procedure is one of the main reasons why culture techniques are widely used, despite their limitations, which have been outlined above (i.e., a large majority of viable bacteria are non-cultivable). This approach produces finely resolved data from a complex environmental sample. Such data will be key in the investigation of the sources of potential pathogens measured at the observation site over long time periods.

### 4.1. Seasonality and Comparisons of the Major Primary Bioaerosols Present at Saclay

This section investigates the variability in time of three major primary bioaerosols—Pollen, fungal spores, and bacteria—Measured in the Total Suspended Particles (TSP) at the same place and the same sampling height. Correlation between different bioaerosols provides insight into possible common sources or atmospheric behavior. This approach was used by the authors of [50], who showed different seasonal variation patterns suggesting that bacteria were associated with particulate matter larger than 10 microns. PM > 10 is an aerosol size fraction, which is rarely reflected in studies that generally consider 10 µm as the upper limit of interest. Yet, PM>10 may significantly contribute to the structure of the entire airborne bacterial community.

**Figure 8:**
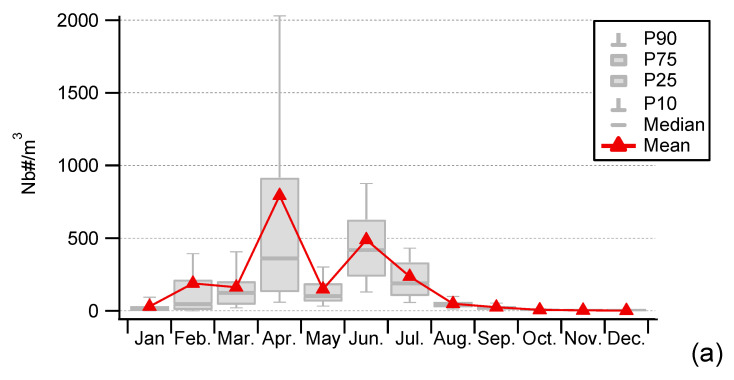

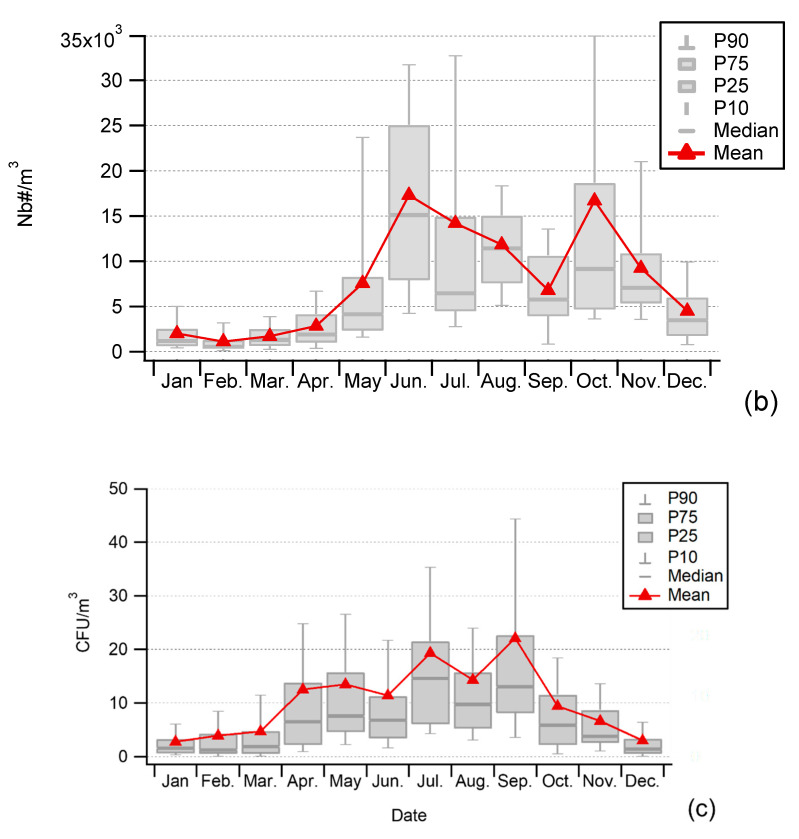
Seasonal variations of Airborne Pollen grains (**a**), Airborne Fungal Spores (**b**), and Airborne cultivable Bacteria (**c**) measured at Saclay observatory from 10 January 18 to 31 May 20.

Figure 8 plots the 12-month seasonal patterns produced by this dataset for bacteria, and from our previous works for pollen and fungal spores [41,42]. Three modes in total are evident. A first mode appears early in spring for bacteria and pollen. A more pronounced mode occurs in summer for all bioaerosols, and then there is a third mode identifiable in autumn, which concerns bacteria and fungal spores only. To our knowledge, according to the scarce literature based on long-term observations, Saclay is the only site reporting a mode early in the spring for bacteria. It might be in relation to the maximum of tree pollens concentrations and the meteorological conditions during the early spring associated with sunny days and described in detail in [41,42]. Briefly, during early spring there is an increase in leaf area (from trees), which provides a favorable biotope for the growth and the multiplication of epiphytic bacteria such as *Pseudomonas syringae*. Afterwards, they can be transported by the wind as well as pollen and subsequental be collected at the Saclay observatory, as illustrated by Figure 7.

We calculated the Annual cultivable Bacteria Integral (ABIn) at Saclay analogous with the Annual Pollen Integral (APin), and Annual Fungal Spores Integral (AFSIn) recommended nomenclature by the authors of [71]. The ABIn calculation resulted in 153.320 CFU/m^3^ for 2018 and 174.915 CFU/m^3^ for 2019 that is a 14% between the periods of observation. The APin and AFSIn for the same periods were −40% and +4%, respectively, which suggests that the meteorological parameters like rain, temperature, and sunshine have a direct effect on annual concentrations of the major atmospheric bioaerosols from one year to the other.

### 4.2. Long-Term Observations and Identification of Potential Sources

Emissions of bacteria from inland surfaces have been estimated to be 30–50 Tg per year, while the emissions by the oceans are several orders of magnitude smaller: 10 mg per year [72,73]. Each biosphere compartment has its specific sources and biological processes which explain such a difference. The regional ecosystem of Saclay is characterized by large forests, crops, and big urban areas nearby.

The early spring peak and the two summer maxima might be linked to regional and continental sources of epiphytic bacteria associated with hardwood areas and largely due to the abundant vegetation coverage that provides for leaf dwelling bacteria [49,74,75]. In this period of the year, continental areas are subject to strong vertical air flux from the ground created by solar heating that can transport communities of small bacteria over long distances mixing all bioaerosol within the air column [76,77]. Bacteria are expected to be eukaryotic species aggregated in clumps attached to pollen, fungal spores, or plant debris. Indeed, our observation of Petri dishes exhibited large and small various colonies regarding the size and the color (Figure A2). This is consistent with previous studies that showed that larger particles contain a large proportion of cultivable bacteria. One explanation could be that these bacteria were not in a spore state but in cell state and could be extracted from the samples via simple vortexing rather than through sonication [78,79].

In autumn and winter, processes of decomposition in the soil increase fungal spore concentrations as rain and even snow conditions intensify. Bacteria are expected to be prokaryotic species originating from the soil, and resuspended by late agricultural activities such as harvesting and then plowing. Well-known plant pathogens, like *Pseudomonas*, can be brought down to the soil together with degraded leaves, thus may contribute to size of colony changes observed in the culture method.

In this context, the air itself is expected to carry other types of bacteria, and the relationship between bacteria and aerosol sources and chemical components is of interest. In particular, fossil fuel and biomass burning emissions are high in cold seasons, and their consumption creates an urban heat island phenomenon that has an artificial positive temperature bias. Among other human activities, sustained road traffic in urban areas favors suspension and potential mixing of prokaryotic bacteria [80]. Such processes may provide new surfaces and material for growing and transporting atmospheric bacteria. At Saclay, very small size of cultivable bacteria was observed to be associated with urban pollution events (Figure A2). This is consistent with [49,67] that reported: “Petri dishes were primarily composed of small, single cultivable bacterial particles which usually occurred during winter.”

**Figure 9:**
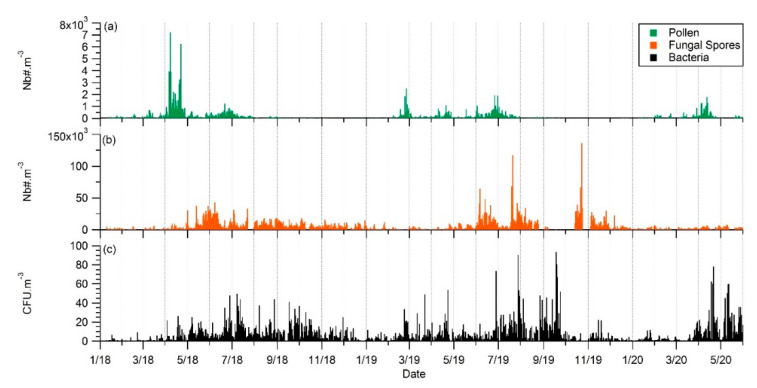
Variability of the three major airborne bioaerosols: Pollen grains (**a**), Fungal Spores (**b**), and cultivable Bacteria (**c**) measured at Saclay observatory from (1 January 2018 to 31 May 2020).

It is interesting to note that the variability in concentration is increasing while the size of the bioaerosols is decreasing (Figure 9) and that bacteria have also previously been positively correlated with the sub-micron size particulate Organic Matter (OM) [28]. It calls for further studies of possible relationships between aerosol chemical composition and the allergenicity or infectivity of some pathogenic bacteria. The role of ammonium nitrate aerosols on the increase of nitrophilic bacteria attached is of particular interest, as the atmosphere is itself a complex microbiome [81,82,83].

### 4.3. Daily Variability, Meteorological Parameters, and Transport

**Figure 10:**
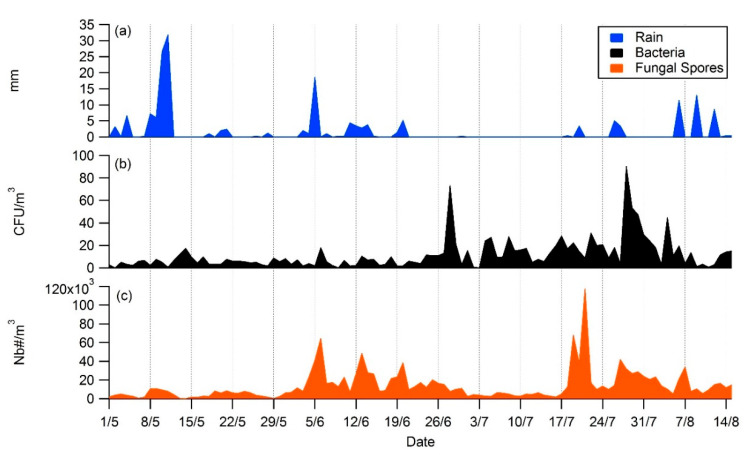
Effect of a rain events (**a**), blue color in mm on atmospheric cultivable bacterial concentrations (**b**),black color in CFU/m^3^ and fungal spores concentrations (**c**), orange color in NB#/m^3^ during summer 2019 at Saclay.

**Figure A4:**
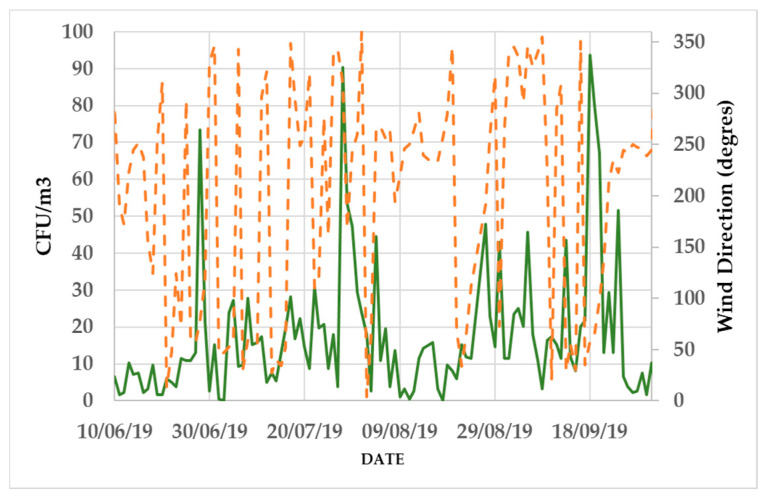
Variability of Wind direction in Degrees° (orange curve) and culturable bacterial concentrations in CFU/m^3^ (green curve) from 10 June 2019 to 23 September 2019.

Rain is often referenced to be a wet removal agent for bioaerosols [84]. However, more recent work suggests that it may act as a trigger for their generation [85]. For instance, in summer 2019, meteorological conditions were anticyclonic: low wind speed and instability in wind direction (Figure A4). On a daily basis, the presence of short showers was negatively correlated with bacterial concentration due to wet deposition, which is consistent with observations made by the authors of [86]. Interestingly, the increase of bacterial concentration and fungal spores concentrations appears often just after the rain episode, as illustrated by Figure 10, which is consistent with observations reported by the authors of [85]. Due to the source dependency and the mechanisms involved in the dry and wet removal processes, also referred to as “splash” dispersal in the literature, the concentrations observed are thought to be locally driven, rather than regionally or transported from large-scale sources during the summer season [74,87,88].

Our observations also suggest that atmospheric concentrations of bacteria are strongly source-dependent and that the role of the rain needs to be better understood. Wind direction, temperature, and rain showers are the main drivers during the summer season, as reported by the authors of [86,89]. In particular, some species undergo strong differences in growth following wet, dry, or “splash” discharges. In order to better understand the effects of rainfall events on bacteria diversity, abundance and dispersal processes, models need to integrate the nature, the type, the abundance and the intensity of the rain shower in relation with the temperature, as well as the dew point data at the finest time and space resolution possible [90,91,92].

**Figure A5:**
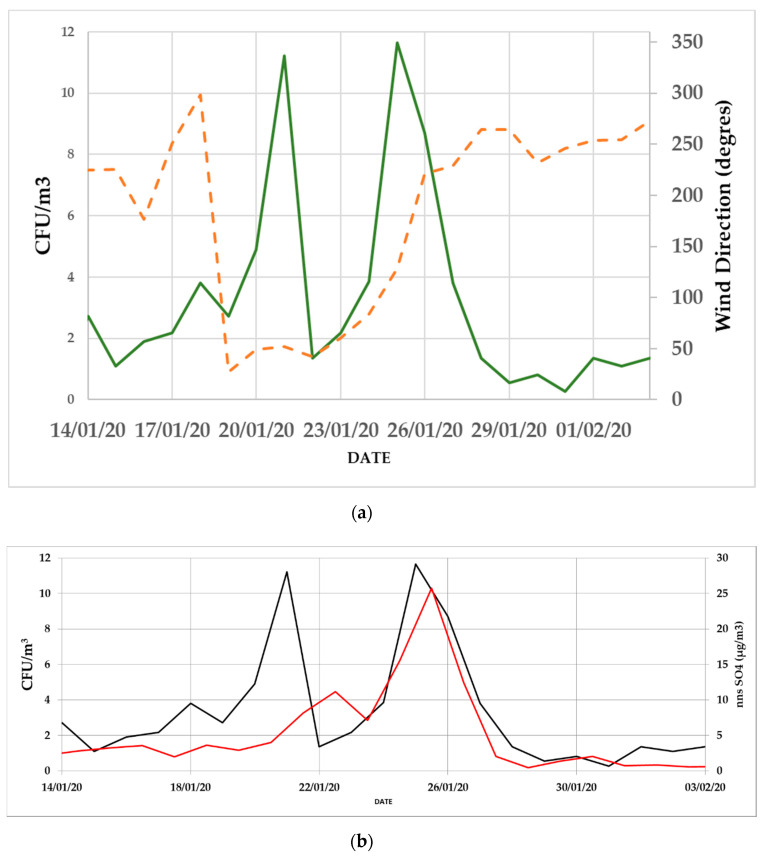
Variability of Wind direction in Degrees° (orange curve) and culturable bacterial concentrations in CFU/m^3^ (green curve) (**a**) and (**b**) variability of non-sea salt sulfate (nss) concentrations in µg/m^3^ (red curve) and culturable bacterial concentrations in CFU/m^3^ (black curve) from 14 January 2020 to 02 March 2020.

To go further in the understanding of the daily variability of the bacterial concentration, we investigated PM1 metrics from the ACSM. Since Saclay is impacted by both regional and continental sources, bacterial concentrations were expected to be transported by different plumes. For example, two equal levels of concentration in January 2020 (10 CFU/m^3^) were associated with wind directions of NW, then N, and finally NE in less than three days. Background bacterial concentrations slowly rose on the 21st, brought by ocean air masses, a first maximum starting the 21th a second relative maximum occurred on the 25th, four days later. During this event, sulfate concentrations at Saclay started to increase on the 22nd (data not shown), indicating that the site was impacted by continental air masses. This episode illustrated how bacteria can be transported from a dense urban area like Paris megacity (first peak attributed to the regional source of Paris) and from continental sources (the second peak, processed air masses) in less than 5 days during the winter period (Figure A5). As recently proposed by the authors of [93], the integration of chemical and biological data in aerosol studies represents a new challenge in atmospheric sciences, and with this perspective, it will be possible to gain a clearer and deeper comprehension of biogeochemical cycles.

### 4.4. Factors Controlling the Seasonal Cycle of Airborne Bacterial Concentrations at Saclay

**Figure 11:**
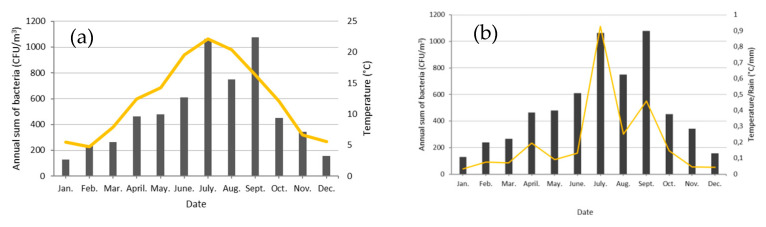
(**a**) Monthly Sum of Bacterial Concentrations (Bars in CFU/m^3^) and Temperature (yellow curve in °C) and (**b**) Monthly Sum of Bacterial Concentrations (Bars in CFU/m^3^) and Temperature/Rain (yellow curve in °C/mm) from 10 January 2018 to 31 March 2020.

**Figure A6:**
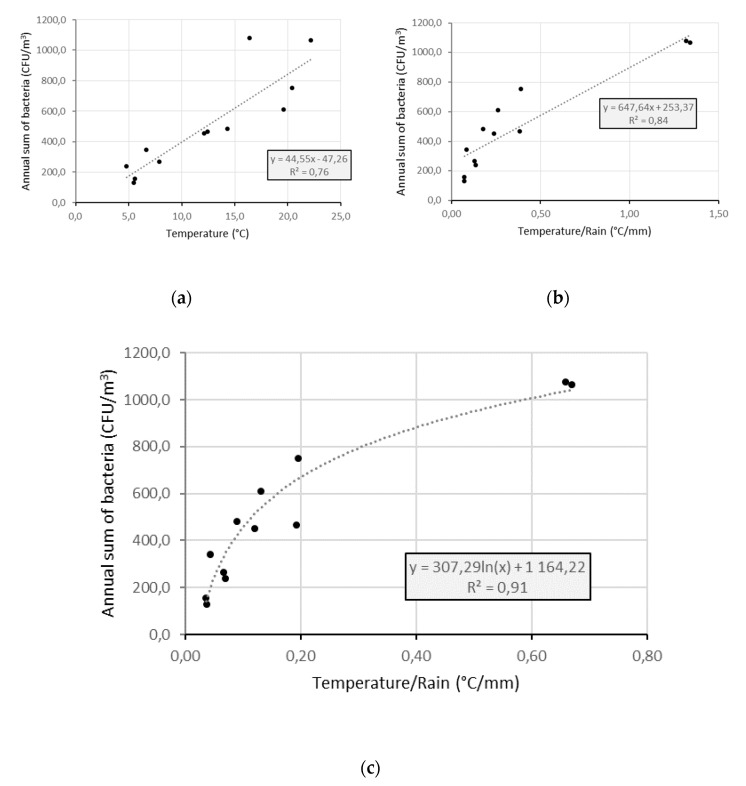
Linear regression of Temperature (°C) and CFU/m^3^ (**a**) and ratio Temperature/Rain and CFU/m^3^ (**b**), logarithmic regression CFU/m^3^ and ratio Temperature/Rain and CFU/m^3^ (**c**).

The literature reports that meteorological parameters such as temperature are a key driver for the increase of atmospheric bacterial concentrations and that ambient PM can serve as support for transport and growth of potential pathogens [94]. Our previous works on pollen and fungal spores [41,42] brought us insights to model the seasonal cycle of bacteria at Saclay. Based on our understanding of the impact of rainfall and temperature on bioaerosol concentrations, at different scales (annual and daily variability), the monthly mean temperature was normalized by the monthly sum of precipitation. We observed that temperature could be considered as the first key factor in seasonality (R^2^ = 0.76, results from Table A2, Figure 11a and Figure A6a). Furthermore, normalizing T (°C) by the sum of Rain (mm) could reasonably reproduce the seasonal cycle of cultivable bacterial concentrations at Saclay with a satisfactory linear correlation coefficient (R^2^ = 0.84, Figure 11b and Figure A6b) and better one by the use of a logarithmic fit (R^2^ = 0.91) and illustrated by Figure 11b and Figure A6c.

In the literature, the wet removal of ambient aerosol particles on short time scales cannot be extrapolated to simulate short or long term observations. It is the differences between the chemistry of inorganics and organics of the coarse mode and bioaerosols. Moreover, the way to analyze the effect of rain on bioaerosol concentrations in stressed environments like the desert and the East part of the Mediterranean is even more complicated. The water provided by rain is a limiting factor for their growth, their re-suspension, and their dispersion.

### 4.5. Seasonal Geographical Origins of Bacteria Concentrations Measured at Saclay

**Figure 12:**
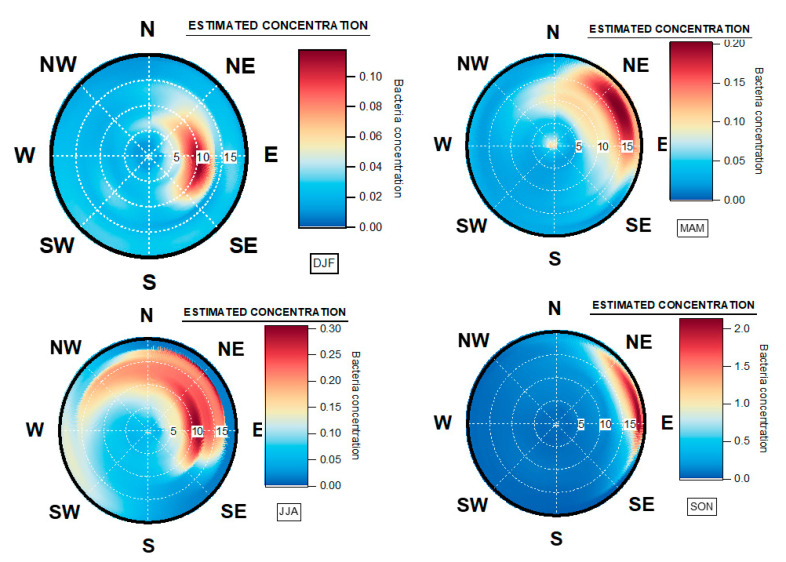
Origin of total atmospheric bacteria using SWIM model Origin by seasons. The dotted white circles represent the wind speed scale in kilometer per hour (km/h). The color grid represents the estimated concentration (Nb#/m^3^) for any wind speed and wind direction. Winter: December–January–February (DJF), Spring: March–April–May (MAM), Summer: June–July–August (JJA), Autumn: September–October–November (SON).

The calculations using ZeFir from this 2-year dataset showed an interesting result regarding the seasonal origin of bacterial concentrations (Figure 12). The source receptor model pointed out that the main origin of bacteria located between the NE and the E sectors are completely independent of the SW prevailing winds (Figure A3), which mostly carries humid marine air masses. During the winter period, from December to February, atmospheric bacteria originated from the E sector, associated with moderate winds from 5 to 12 km/h. During the spring season, from March to May, the geographical origin was clearly from NE, associated with wind speeds in the range of 15 km/h, indicating long-range transport. Regional sources were also evidenced within the N to E sectors.

In summer, model outputs indicate bacteria inputs from NW to SE, associated with wind speeds from 5 to 15 km/h. This season is expected to exhibit more biodiversity from marine, urban and inland species. In autumn, the origin is from NE to E with wind speeds from 15 to 20 km/h, indicating long-range transport.

Therefore, the origin of atmospheric bacteria measured at Saclay is mostly continental, which is in accordance with what has been reported by the authors of [23,24]. The results of the model agree very well with what has been observed by the authors of [95,96], both in terms of seasonality and of daily variability. It underlines the strong capacity of the surrounding ecosystem to generate epiphytic and telluric bacteria (see Figure A2). As a consequence, the results obtained suggest that the expected biodiversity at Saclay will be correlated to specific origins, but also mixing processes, as seasonal change, like vertical mixing in summer, reduced wind speed and bioaerosol transport [77]. This last point could have direct implications on the formation of the cloud cover, through their CCN and IN properties, and the presence of summer thunderstorms. As described by the authors of [97], the atmosphere can be considered as a strong “washing machine,” which mixes all sources from bottom to top, and back to bottom. Recent works have highlighted that enrichment of bacterial concentration in the PM2.5 could induce inflammation on allergic organisms [98]. Interestingly, the allergic response followed by IgE from humans, in the megacity of Paris, may be linked to the presence of some bacteria on aeroallergens (Pascal Poncet, Institut Pasteur personal communication).

## 5. Conclusions

In the literature, approaches other than the culture technique have been used to quantify the concentration of atmospheric microorganisms. Our study reports the bacterial atmospheric concentrations using the cultivable method for the purpose of (1) developing an alternative analysis method based on flow cytometry technique to estimate the total bacterial concentration in the air, (2) understanding the atmospheric variability of cultivable bacterial concentration impacting a suburban area of Paris and (3) applying a source receptor model in an attempt to identify their geographical origins. Several cases studies served to investigate the link between bacterial concentration compared with other parameters like pollen, fungal spores, meteorological data, or pollution events. The seasonal variation of bacterial concentration exhibits a tri-modal pattern that was found in April-May, July, and September. The lowest concentrations were found to be in winter. The tri-modal pattern was thought to be in relation to the seasonal cycle of pollen and fungal spores. The daily variability was found to be mainly driven by wind direction, temperature, and rain showers. Surprisingly, in winter, bacteria can be transported from dense urban cities and over long distances if they are attached to other particles. Our study is based on more than 30 cultivable atmospheric bacteria, among which, the majority was expected to be eukaryotic rather than prokaryotic. Bacteria typically originated from NE to E direction, brought by moderate winds. Their sources evolved from continental to regional and local from winter to summer. At this stage of our study, the biodiversity have been analyze by analyzed by PCR (Polymerase Chain Reaction) techniques to amplify the 16S rRNA (RiboNucleic Acid) gene followed by sequencing methods in order to characterize the biodiversity of the bacterial community, under different air mass regimes. The results are under interpretation and they will be compared with the culture and flow cytometry techniques in a companion paper. As suggested by many authors, the bioaerosol science community now needs long-term observations to better understand impacts of the land-use changes, the reduction of anthropogenic emission and the effect of the global warming on airborne microorganisms. concentrations

## Figures and Tables

**Table 1:** Description of the procedure used for measurements with the flow cytometer Attune NxT.

Actions	Steps	Function
Performance Test	1	Internal Instrument Verification
Sanitize	2	Microfluidic System Cleaning
5 µm Filtered Bacteria Sample with SY9	3	Verifications or Adjustment of the PM
Sanitize	4	Microfluidic System Cleaning
Ultra-Pure Water with SY9	5	Identification of the Background
Sanitize	6	Microfluidic System Cleaning
5 µm Filtered Atmospheric sample with SY9	7	Identification and counting of total bacteria
Sanitize	8	

## Appendix A

**Table A1:** Total Bacteria counted in the 3 mL sample from the extraction of the atmospheric samples where TB represents the Total Bacteria, R1, R2, R3, R4, R5 represent the different populations gates identified.

Sample	Date	TB	R1	R2	R3	R4	R5	TC CFU/m^3^	R3 CFU/m^3^	LOSSES%
N408	21 February 19	240	12,000	381,300	31,800	2400	1200	4	578	99.2
N409	22 February 19	1850	34,500	553.8	46,800	450	450	34	851	96.0
N410	23 February 19	580	7200	219,600	21,000	3900	5280	11	382	97.2
N411	24 February 19	1150	7200	276,600	30,300	3000	13,500	21	551	96.2
N412	25 February 19	1190	33,240	701,259	69,141	6321	20,760	22	1257	98.3
N413	26 February 19	760	41,940	563,820	55,839	5241	3921	14	1015	98.6
N414	27 February 19	540	36,699	486,519	33,399	4761	9159	10	607	98.4
N415	28 February 19	300	9339	73,860	5241	1419	2520	5	95	94.3
N416	1 March 19	130	2460	94,101	4059	960	381	2	74	96.8
N417	2 March 19	150	1461	50,061	1959	1599	1599	3	36	92.3
N418	3 March 19	100	8799	54,639	1821	1920	2541	2	33	94.5
N419	4 March 19	200	4641	30,561	1320	741	921	4	24	84.8
N420	5 March 19	460	4140	43,401	1980	180	180	8	36	76.8
N423	8 March 19	20	2001	20,739	1161	381	420	3	21	86.2
N424	9 March 19	110	1680	31,581	2601	201	159	0	47	99.2
N425	10 March 19	420	6501	30,120	1311	459	141	2	24	91.6

**Table A2:** Monthly means of Rain (mm), Temperature (°C) and sum of cultivable bacteria (CFU/m^3^) from 1 January 2018 to 31 May 2020.

Month	Rain (mm)	T (°C)	CFU/m^3^	T/Rain (°C/mm)
January	167.1	5.5	128.8	0.07
February	63.7	4.8	238.2	0.14
March	113.3	7.9	265.6	0.13
April	63.8	12.5	465.2	0.38
May	157.5	14.3	480.4	0.18
June	148	19.6	608.9	0.26
July	23.9	22.2	1064.0	1.34
August	81.7	20.4	750.8	0.39
September	35.8	16.4	1077.3	1.32
October	82.6	12.1	452.1	0.24
November	142.9	6.7	342.7	0.09
December	133.9	5.6	155.3	0.07
